# Coadministration of the Three Antigenic *Leishmania infantum* Poly (A) Binding Proteins as a DNA Vaccine Induces Protection against *Leishmania major* Infection in BALB/c Mice

**DOI:** 10.1371/journal.pntd.0003751

**Published:** 2015-05-08

**Authors:** Manuel Soto, Laura Corvo, Esther Garde, Laura Ramírez, Virginia Iniesta, Pedro Bonay, Carlos Gómez-Nieto, Víctor M. González, M. Elena Martín, Carlos Alonso, Eduardo A. F. Coelho, Aldina Barral, Manoel Barral-Netto, Salvador Iborra

**Affiliations:** 1 Centro de Biología Molecular Severo Ochoa (CSIC-UAM), Departamento de Biología Molecular, Universidad Autónoma de Madrid, Madrid, Spain; 2 LeishmanCeres Laboratory (GLP Compliance Certified), Parasitology Unit. Veterinary Faculty, University of Extremadura, Cáceres, Spain; 3 Departamento de Bioquímica-Investigación, Instituto Ramón y Cajal de Investigación Sanitaria (IRYCIS), Hospital Ramón y Cajal, Madrid, Spain; 4 Programa de Pós-Graduação em Ciências da Saúde: Infectologia e Medicina Tropical, Faculdade de Medicina, Universidade Federal de Minas Gerais, Belo Horizonte, Minas Gerais, Brazil; 5 Departamento de Patologia Clínica, COLTEC, Universidade Federal de Minas Gerais, Belo Horizonte, Minas Gerais, Brazil; 6 Centro de Pesquisas Gonçalo Moniz (Fundação Oswaldo Cruz-FIOCRUZ), Salvador, Bahia, Brazil; 7 Immunobiology of Inflammation Laboratory, Department of Vascular Biology and Inflammation, Centro Nacional de Investigaciones Cardiovasculares (CNIC), Madrid, Spain; University of Texas Medical Branch, UNITED STATES

## Abstract

**Background:**

Highly conserved intracellular proteins from *Leishmania* have been described as antigens in natural and experimental infected mammals. The present study aimed to evaluate the antigenicity and prophylactic properties of the *Leishmania infantum* Poly (A) binding proteins (LiPABPs).

**Methodology/Principal Findings:**

Three different members of the LiPABP family have been described. Recombinant tools based on these proteins were constructed: recombinant proteins and DNA vaccines. The three recombinant proteins were employed for coating ELISA plates. Sera from human and canine patients of visceral leishmaniasis and human patients of mucosal leishmaniasis recognized the three LiPABPs. In addition, the protective efficacy of a DNA vaccine based on the combination of the three *Leishmania* PABPs has been tested in a model of progressive murine leishmaniasis: BALB/c mice infected with *Leishmania major*. The induction of a Th1-like response against the LiPABP family by genetic vaccination was able to down-regulate the IL-10 predominant responses elicited by parasite LiPABPs after infection in this murine model. This modulation resulted in a partial protection against *L*. *major* infection. LiPABP vaccinated mice showed a reduction on the pathology that was accompanied by a decrease in parasite burdens, in antibody titers against *Leishmania* antigens and in the IL-4 and IL-10 parasite-specific mediated responses in comparison to control mice groups immunized with saline or with the non-recombinant plasmid.

**Conclusion/Significance:**

The results presented here demonstrate for the first time the prophylactic properties of a new family of *Leishmania* antigenic intracellular proteins, the LiPABPs. The redirection of the immune response elicited against the LiPABP family (from IL-10 towards IFN-γ mediated responses) by genetic vaccination was able to induce a partial protection against the development of the disease in a highly susceptible murine model of leishmaniasis.

## Author Summary

Leishmaniases are a wide spectrum of parasite diseases caused by the infection of different species of *Leishmania* genus in several mammalian hosts, such as humans and dogs, among others. The induction of antibody responses against some parasite intracellular proteins implicated in different structural and functional cellular roles has been associated with parasite survival, infection progression, down-regulation of the cellular immune responses against the parasites and the development of pathology. The data presented in this work show the antigenic nature of the three members of the *Leishmania infantum* Poly (A) Binding Protein (LiPABP) family in different forms of natural and experimental leishmaniasis. Using a susceptible model of progressive leishmaniasis (BALB/c mice infected with *Leishmania major*), it was shown that the administration of a LiPABPs based combined genetic vaccine was able to induce a partial protection against the disease. Redirection of the IL-10 mediated responses elicited by the parasite LiPABPs after *L*. *major* infection towards an IFN-γ specific production by vaccination was related to protection. The inclusion of the LiPABPs in a protective vaccine formulation against leishmaniasis is discussed.

## Introduction

Parasites of the genus *Leishmania* are protozoan organisms that can infect different mammalian species including humans and dogs. *Leishmania* promastigote forms present in the insect vectors (phlebotomine sand flies) are transmitted to the vertebrate hosts during the blood meal [1]. After infection, parasites differentiate in the amastigote forms that multiply inside macrophages. The infected vertebrates can present a wide spectrum of clinical manifestations, globally known as leishmaniasis. Depending on the host and the parasite species, the disease can range from subclinical forms to severe symptomatic leishmaniasis [2]. In humans, there are various parasite species that cause cutaneous leishmaniasis (CL), including *Leishmania major* in the Old World and *Leishmania braziliensis* in the New World, among other species. CL is the less severe form of the disease and, in some cases, patients heal spontaneously. A percentage of the patients infected with *L*. *braziliensis* can develop a form of the disease characterized by the presence of lesions in mucosal areas, known as mucosal leishmaniasis (ML) [3]. Visceral leishmaniasis (VL) is the most severe clinical form of the disease and it is presented in human and canine patients infected with *Leishmania infantum* (syn. *Leishmania chagasi* [4]) in the Mediterranean basin and in South America, or in human patients infected with *Leishmania donovani* in Asia and Africa [5]. VL patients usually present hepatosplenomegaly accompanied by other clinical symptoms like fever, polyclonal hypergammaglobulinemia and anaemia [6]. Infected dogs develop similar systemic symptoms accompanied by cutaneous lesions in the mucous membranes, skin and eyes [7].

The control of infection in mammalian hosts naturally or experimentally infected with different *Leishmania* species depends mainly on the induction of cellular responses able to activate macrophages to eliminate the intracellular pathogens [8]. The induction of CD4^+^ Th1 cells specific for parasite antigens is critical to control infection due to their ability to secrete IFN-γ, but the activation of other cell subsets to produce this cytokine, like *Leishmania* antigen specific CD8^+^ T cells, is also required for a protective host response to *L*. *major* infection in the C57BL/6 mice strain. On the contrary, the generation of an IL-4 driven Th2 response after *L*. *major* infection in the susceptible BALB/c mice strain has been related to disease (reviewed in [9]). In this model of murine leishmaniasis, T cell or B cell derived IL-10 production has also been implicated in the progression of infection [10,11].

The fact that patients recovered from the disease are resistant to *Leishmania* reinfection can be taken as an indication that a vaccine is feasible. Regarding prophylaxis in dogs, there are three commercial vaccines against canine leishmaniasis: Leishmune, based on the Parasite Fucose-Manose-Ligand [12], Leish-Tec, a vaccine constructed by the recombinant version of the amastigote specific A2 protein [13] and CaniLeish, composed of promastigote secreted-excreted factors [14], but none of them are able to completely control the disease [15,16]. In addition, there is no vaccine against this parasite in humans. Some of the candidates for the development of human vaccines have been tested as second generation products (based on parasite or insect vector saliva defined antigens) or third generation DNA based vaccines in murine experimental models [17]. The establishment of a protective anti-*Leishmania* response based on these recombinant molecules requires the induction of parasite specific long-lasting memory T cells that will expand as effector T cells for the production of IFN-γ dependent responses specific for parasite antigens shortly after infection. Some experimental vaccines based on intracellular proteins that form complexes with nucleic acids, like histones or ribosomal proteins have been related with protection against the disease in different experimental models [18,19,20,21,22,23].

Poly(A) binding proteins (PABPs) are nuclear or cytosolic factors that contribute to mRNA metabolism from nuclear polyadenylation and export to translation initiation and control of RNA turnover in the cytoplasm [24]. PABPs are able to interact with the Poly(A) tail and other regions of the mRNAs as well as with other protein factors to exert their functions [25]. Three members of the PABP family have been described in *L*. *major*. Two of them, namely LmPABP1 and LmPABP2, are the cytosolic or nuclear (respectively) orthologues of the PABPs from other kinetoplastids, whereas the third one (LmPABP3) is a nuclear protein only present in the *Leishmania* genus among kinetoplastids [26]. Since the *L*. *infantum* PABP2 was recognized by the sera from VL symptomatic dogs [27] this work has extended the analysis of the antigenicity of all the members of the LiPABP family. The results of this work have demonstrated the antigenicity of the three LiPABPs in human and canine leishmaniasis. In addition, their immunogenicity and protective capacity when administered as a third generation DNA combined vaccine have been evaluated in a susceptible experimental model of murine leishmaniasis: BALB/c mice infected with *L*. *major*.

## Methods

### Mice and parasites

Female BALB/c mice (6–8 weeks old) were purchased from Harlan (Barcelona, Spain). All procedures were performed according to the Directive 2010/63/UE from the European Union and RD53/2103 from the Spanish Government and approved by the Animal Care and Use Committee at the Centro de Biología Molecular Severo Ochoa (reference CEEA-CBMSO 21/138) and the Bioethical Committee of the Consejo Superior de Investigaciones Científicas (reference 100/2014). The project was finally authorized by the Government of the Autonomous Community of Madrid (reference PROEX121/14).

Promastigotes of *L*. *infantum* (MCAN/ES/1996/BCN/150/MON-1) and from *L*. *major* clone V1 (MHOM/IL/80/Friedlin) were cultured at 26°C in Schneider medium (Gibco, NY, U.S.A.) supplemented with 10% Fetal Calf Serum (FCS) (Sigma, MO, U.S.A.), 100 U/ml of penicillin and 100 μg/ml of streptomycin. Parasites were kept in a virulent state by passage in BALB/c mice. *L*. *major* amastigotes were obtained from infected popliteal lymph nodes. After transformation to the promastigote form, parasites were grown until stationary phase and then harvested for inoculation in the left hind footpad of mice or for Soluble *Leishmania* Antigen (SLA) preparation as described in [22].

### Cloning of DNA sequences coding for *L*. *infantum* PABPs and recombinant products manufacturing

The *L*. *infantum* PABP1 (LinJ35.V3.5360), PABP2 (LinJ.35.4200) and PABP3 (LinJ.25.0080) (namely LiPABP1, LiPABP2 and LiPABP3, respectively) coding regions were obtained from the *L*. *infantum* genome database (www.genedb.org) using the *L*. *major* orthologous protein sequences as probes (LmjF.35.5040, LmjF.35.4130 and LmjF.35.4130) [26]. Coding regions were PCR amplified using genomic DNA from *L*. *infantum* (taking advantage of the lack of intron sequences in this genus) and the next specific primers: LiPABP1 forward, 5’-CGGGATCCATGGACAAGCCGATGCAGATC-3’ and reverse, 5’-CGGATATCACGCCGTCTGATGCGCCTT-3’; LiPABP2 forward, 5′- CGGGATCCATGGCCTTTACTGGTCCGAATC-3′ and reverse, 5’-CGGGATCCTCAAACGCTCATGTCTGCCAAG-3’; LiPABP3 (forward, 5’-CGGGATCCATGGTGGTCCCAGTGCAACG-3; reverse, 5’-CGGATATCAGTTGCCAGTGTGCTGCTGAAG-3’. The restriction sites included for cloning purposes (*BamHI* for LiPABP2 and *BamHI/EcoR*V for LiPABP1 and LiPABP3) are underlined. The resultant PCR products were inserted into pBluescript II SK (Stratagene. CA, U.S.A). For that purpose the PCR inserts were purified using the High Pure PCR Product Purification Kit (Roche Diagnostics GmbH, Mannheim, Germany) and the pBluescript II SK and the PCR inserts were digested with the indicated restriction enzymes (New England Biolabs, MA, U.S.A.) and ligated with the T4 DNA ligase (New England Biolabs). Positive clones were selected by a restriction analysis (using the enzymes employed for cloning) and double stranded sequenced. For subcloning purposes, the three inserts containing the coding regions were obtained by digestion with *BamH*I for LiPABP2 and *BamH*I/*EcoR*V for LiPABP1 and LiPABP3, and inserted in two different plasmids. For preparing genetic vaccines, the inserts were subcloned in the corresponding sites of the pcDNA3 mammalian expression vector (Invitrogen, CA, U.S.A.). The correct orientation of the LiPABP2 insert was monitored by a restriction analysis made with *Apa*I, an enzyme that cut the LiPABP2 coding region (positions 1651–1656 of a total length of 1756 nucleotides; Gene DB sequence LinJ.35.4200) and it is included in the multiple cloning site of the pcDNA3 plasmids downstream the *Bam*HI cut site. For vaccination assays, the pcDNA3 vector and the three recombinant pcDNA3 plasmids were purified by the endotoxin-free Giga-preparation Kit (Qiagen, Hilden, Germany). Plasmids used in vaccination assays were suspended in sterile phosphate-buffered saline (PBS). For recombinant protein preparations, DNA inserts containing the different coding regions were subcloned into the *BamH*I (LiPABP2) or the *BamH*I/*Sma*I sites (LiPABP1 and LiPABP3) of the pQE30 prokaryotic expression vector (Qiagen). The correct LiPABP2 insert orientation was examined by restriction with *Sma*I enzyme, since the LiPABP2 coding region has a *Sma*I cut site located at nucleotides 1173–1178 of a total length of 1756 (Gene DB sequence LinJ.35.4200) and it is also located downstream the *BamH*I cut sequence in the pQE multiple cloning site. *Escherichia coli* (M15 strain) transfected with the recombinant plasmids were employed for over-expression of the his-tagged proteins. For that purpose, *E*. *coli* cultures were grown at 37°C in an orbital shaker to the mid-growth phase (determined by 0.6 value of Optical Density (O.D.) at 600 nm) and the expression was induced by addition of 1 mM IPTG for 4 h at the same conditions. Since the three LiPABPs formed inclusion bodies, proteins were solubilized under denaturing conditions. For ELISA assays, proteins were purified under denaturant conditions onto Ni-nitrilotriacetic-acid-agarose (Ni-NTA, Qiagen) columns following the manufacturer’s instructions (The QIAespressionist^TM^; https://www.qiagen.com/es/resources/resourcedetail?id=79ca2f7d-42fe-4d62-8676-4cfa948c9435&lang=en). Briefly, proteins were solubilized in binding buffer (20 mM Tris ClH pH 8.0, 0.5 M NaCl, 8 M Urea, 1 mM β-mercaptoethanol) supplemented with 5 mM Imidazole, and mounted into a Ni-NTA gravity flow column. After washing in the binding buffer supplemented with 20 mM imidazole, proteins were eluted using the binding buffer supplemented with 0.5 M imidazole. After, purified proteins were dialyzed against ELISA denaturant coating buffer (20 mM Tris ClH pH 8.0, 0.5 M NaCl, 3 M Urea, 1 mM β-mercaptoethanol). For immunization or cells culture experiments, proteins were purified under denaturing conditions and refolded on the affinity column as described in [28] adding 20% (p/v) glycerol in those solutions that had not the denaturant agent (urea) to maintain protein solubility [29]. After purification, proteins were dialyzed against PBS plus 20% (p/v) glycerol. Polymyxin-agarose columns (Sigma) were employed to remove residual endotoxin content (<10 pg of LPS per μg of recombinant protein, measured by the Quantitative Chromogenic Limulus Amebocyte Assay QCL-1000 (BioWhittaker, MD, U.S.A.)). Protein concentration was estimated by Bradford method using the Bio-Rad Protein assay (Bio-Rad, CA, U.S.A.).

### Sera and ELISA assays

Human serum samples were obtained from clinical and parasitologically diagnosed patients. For the present study, anonymized samples were randomly selected from a sera bank (LIP-CPqGM-FIOCRUZ) built for previous independent studies conducted in Brazil in endemic areas for ML [30] or VL [31]. Canine symptomatic VL sera from *L*. *infantum* infected dogs were collected in the Extremadura region of Spain [32]. Control sera were obtained from healthy individuals (humans or dogs). For ELISA, MaxiSorp plates (Nunc, Roskilde, Denmark) were coated with 0.2 μg of each one of the recombinant antigens (diluted in ELISA denaturant coating buffer) for 12 h at 4°C. After four washes with PBS + 0.5% Tween 20 (PBST) wells free binding sites were blocked with blocking solution (PBST + 5% non-fat milk solution) for 1 h at room temperature (RT) and incubated with human or canine sera for 2 h at RT (1/200 dilution in blocking solution). After four washes in PBST, wells were incubated with secondary antibodies conjugated to horseradish peroxidase at 1/2,000 dilution in blocking solution for 1 h at RT (anti-dog IgG or anti-human IgG from Nordic Immunological Laboratories, Tilburg, The Netherlands). After four washes in PBST, the reaction was developed through incubation with orto-phenylenediamine 10 min in the dark, and stopped by addition of 2 N H_2_SO_4_. Optical densities were read at 450 nm in an ELISA microplate spectrophotometer (Bio-Rad).

From the experimental model, sera were obtained at the beginning of the immunization (pre-immune sera), four weeks after the last doses (just before challenge with parasites) as well as seven weeks after challenge with *L*. *major*. The procedure for the ELISAs were the same indicated above but the reciprocal end-point titer (defined as the inverse of the highest serum dilution factor giving an absorbance > 0.15) against the recombinant proteins (0.2 μg per well) or SLA (1 μg per well) was determined by serial dilution of the sera. Anti-IgG, anti-IgG1 or anti-IgG2a (all of them at 1/2,000) horseradish peroxidase-conjugated anti-mouse immunoglobulins were used as secondary antibodies (purchased by Nordic Immunological Laboratories).

### Expression of LiPABPs in mammalian cells

To confirm that the DNA constructs were functional, COS7 cells were independently transfected with 20 μg of pcDNA3-LiPABP1, pcDNA3-LiPABP2 or pcDNA3-LiPABP3 using the Fugene reagent (Promega, WI, U.S.A.) according to the manufacturer's protocol. Seventy-two hours post-transfection, the cells were harvested, washed two times with ice-cold PBS, and immediately lysed by the addition of Laemmli's buffer. The proteins were resolved by sodium dodecyl sulfate-polyacrylamide gel electrophoresis (SDS-PAGE) and transferred to nitrocellulose membranes (Amersham, Aylesbury, United Kingdom). The blots were probed with mouse antiserum raised against the LiPABPs (1/200) by the independent immunization of the recombinant LiPABPs (three doses, 12 μg of each one) combined with 25 μg of the next two phosphorothioate-modified CpG-ODNs: (5’-TCAACGTTGA-3’ and 5’- GCTAGACGTTAGCGT-3’). Anti-IgG (1/2,000) horseradish peroxidase-conjugated anti-mouse immunoglobulins were employed as secondary antibodies (Nordic Immunological Laboratories). As control, similar blots were probed with an anti-human PABP antibody (Cell Signaling, Merck Millipore Darmstadt, Germany) and horseradish peroxidase-conjugated anti-mouse immunoglobulins (1/2,000; Nordic Immunological Laboratories) as secondary reagent. Western blots treated with anti-LiPABPs sera were revealed with the ECL Western Blotting System (GE Healthcare Life Sciences, PA, U.S.A.) and those probed with anti-human PABP were revealed with 4-chloronaphthol (Sigma).

### Immunization, parasite challenge and parasite quantification

Three mice groups (n = 16 per group) were made. The LiPABP vaccinated group was inoculated in the right hind footpad with 200 μg of a mixture of the next recombinant plasmids: pcDNA3-LiPABP1, pcDNA3-LiPABP2 or pcDNA3-LiPABP3 (66.6 μg of each plasmid). As control groups, mice were inoculated with PBS or with 200 μg of the pcDNA3 plasmid. Mice were inoculated three times (two weeks apart). Four weeks after last dose eight mice per group were euthanized, in order to obtain their spleens to be used in the analysis of the cellular immune responses induced by immunizations. Parasite challenge was carried out at the same time using the remaining mice by subcutaneous inoculation with 5 × 10^4^ stationary-phase promastigotes of *L*. *major* into their left footpads. For the analysis of the clinical signs of infection, footpad swelling was measured with a metric caliper (expressed as thickness of the infected left footpad minus thickness of the right footpad). For parasite load determination, the spleen and the single popliteal lymph node draining the site of infection (left leg) were collected and independently processed. The complete spleens or lymph nodes were mechanically homogenized in Schneider's medium supplemented with 20% heat-inactivated FCS, 200 U/ml penicillin and 100 μg/ml streptomycin and filtered using a cell strainer (70-μm pore size). Each homogenized sample tissue was serially diluted (1/3) in a 96-well flat-bottomed microtiter plate containing the same medium (in triplicates). The number of viable parasites was determined from the highest dilution at which promastigotes could be grown up to 10 days of incubation at 26°C as previously described [33].

### Cytokine production

Spleen cells obtained from mice inoculated with saline, with the pcDNA3 vector or with the LiPABPs combined genetic vaccine were seeded and independently cultured in RPMI complete medium at 5 × 10^6^ cells per ml (RPMI medium (Sigma)) supplemented with 10% heat-inactivated FCS, 20 mM L-glutamine, 200 U/ml penicillin, 100 μg/ml streptomycin and 50 μg/ml gentamicin, during 72 h at 37°C in 5% CO_2_. Mice (n = 8 per group) were euthanized four weeks after the last dose or seven weeks after parasite challenge. Cells were cultured alone or with some of the next stimuli: SLA, the recombinant LiPABP1, LiPABP2, LiPABP3 proteins assayed separately (all of them at 12 μg/ml of final concentration), or with a mixture of the three recombinant LiPABPs at 4 μg/ml each one. The levels of IFN-γ, IL-10 or IL-4 were determined in the culture supernatants by sandwich ELISA using monoclonal antibodies specific for mouse cytokines (capture and detection) provided in commercial kits (Pharmingen, San Diego, CA, USA), following the manufacturer’s instructions.

### Statistical analysis

Statistical analysis was performed using the Graph-Pad Prism program. Data were first analyzed by the D'Agostino & Pearson normality test. Parametric data were analyzed by a two-tailed Student's *t*-test and non-parametric data were analyzed by a Mann Whitney test. For both test differences were considered significant when *^(+)^ *P* < 0.05, **^(++)^ *P* < 0.01 or ***^(+++)^ *P* < 0.001.

## Results

### Antigenicity of the *Leishmania* PABPs during natural infections

**Fig 1:**
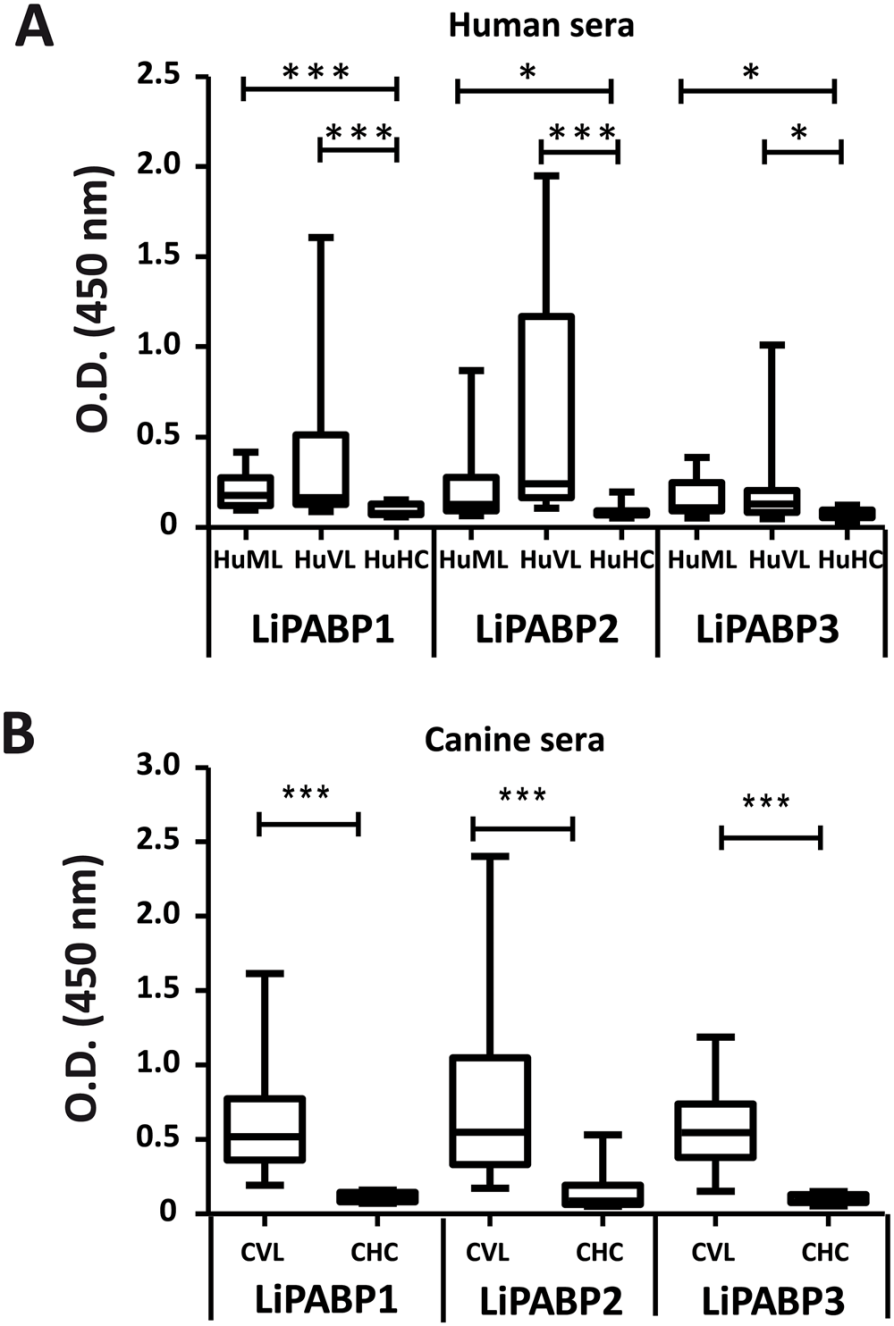
The LiPABPs are antigenic in humans and dogs suffering leishmaniasis. Antibody response of human ML (n = 20) or VL patients (n = 20) and healthy individuals (HC, n = 8) against the three recombinant LiPABP proteins (A). Antibody responses of canine VL (VL, n = 38) and healthy animal sera (HC, n = 18) against the recombinant proteins (B). All sera were tested for IgG reactivity by ELISA (1/200). Horseradish peroxidase-conjugated anti-human IgG (1/2,000) or anti-dog IgG (1/2,000) antibodies were used as the secondary reagents. Results are shown as whisker (min to max) plots. * (P < 0.05) and *** (P < 0.001) statistical differences in the IgG reactivity values between each group of patients and their corresponding healthy control sera group, evaluated by the Mann-Whitney test.

First, the antigenicity of the members of the *Leishmania* PABP was determined. For this purpose, the three LiPABPs were expressed as recombinant proteins in *E*. *coli*, purified by affinity chromatography and employed for ELISA to investigate the presence of circulating antibodies against them in the sera from human and canine patients affected by VL. The three LiPABPs were recognized by Brazilian human patients suffering VL due to the infection of *L*. *chagasi* (syn. *L*. *infantum*) (Fig 1A), and by sera samples from Spanish VL symptomatic dogs infected by *L*. *infantum* (Fig 1B). On the basis of the high degree of identity observed between *L*. *infantum* and *L*. *braziliensis* counterparts (S1 Fig) the cross reactivity between LiPABPs and sera from patients affected by ML due to *L*. *braziliensis* was assayed. The reactivity of the sera from ML patients was significantly higher than that from healthy donors for the three LiPABPs (Fig 1A). From these data, it was concluded that the three members of the LiPABP family interact with the host immune system after infection in different vertebrate hosts.

### Immunogenicity of LiPABPs administered as a combined DNA-vaccine

**Fig 2:**
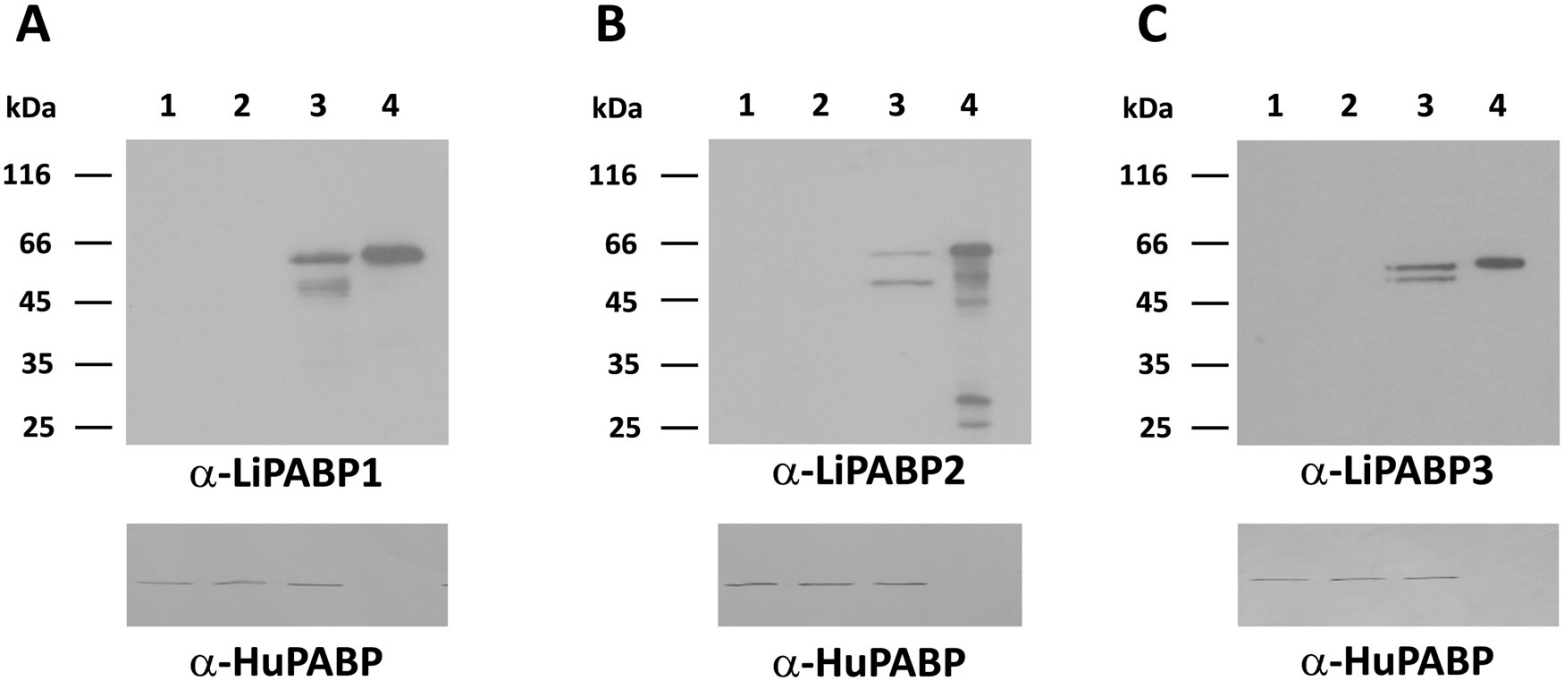
Analysis of the expression of the *L*. *infantum* genes coding for the three LiPABPs in mammalian cells. SDS-PAGE in which 10 μg of total proteins obtained from a COS cell culture (lane 1), COS cell transiently transfected with the pcDNA3 eukaryotic expression vector (lane 2) or cell lines transfected with DNA plasmids pcDNA3-LiPABP1 (A), pcDNA3-LiPABP2 (B) or pcDNA3-LiPABP3 (C) (lanes 3) were resolved. Two μg of the corresponding recombinant proteins expressed in *Escherichia coli* were resolved in the lane 4 of the different gels. After blotting, the expression of the three proteins in the transfected cultures protein was monitored by the detection of the parasite LiPABPs using specific antiserum against LiPABPs obtained from mice immunized with the corresponding recombinant proteins (1/200) (Upper panels). Horseradish peroxidase-conjugated anti-mouse IgG antibodies (1/1,000) were employed as the secondary reagent. Chemiluminescence Western blot detection of each panel is shown. Similar blots were incubated with an anti-human antibody (1/2,000) to reveal the presence of the mammalian PABP (lower panels). In this case, horseradish peroxidase-conjugated anti-rabbit IgG antibodies (1/2,000) were used. Blots revealed with 4-cloronaphtol are shown.

To evaluate the immunogenicity of the *Leishmania* PABPs administered as a combined DNA vaccine, the coding regions for the LiPABPs (LiPABP1, LiPABP2 and LiPABP3) were individually cloned into the eukaryotic expression vector pcDNA3. The correct expression of the DNA vaccines was tested in culture analyzing the expression of the LiPABPs in COS7 cells transfected with the recombinant plasmids (Fig 2, upper panels). Using sera from mice immunized with the recombinant versions of the parasite LiPABPs, specific protein bands were observed in the three immunoblot panels in the lanes corresponding to cell cultures transfected with the recombinant plasmids (Fig 2, upper panels, lanes 3). No reactivity was observed against proteins from COS7 cell samples un-transfected (Fig 2, upper panels, lanes 1) or transfected with the non-recombinant plasmid (Fig 2, upper panels, lanes 2). The presence of the mammalian PABP was revealed by probing similar blots with an anti-human PABP antibody (Fig 2, lower panels). The molecular weights of the parasite LiPABPs expressed by mammalian cells were close to the expected (hypothetically calculated from their amino acid sequences) and slightly lower to that observed for the recombinant LiPABPs expressed in bacteria and possessing a histidine tag in the amino-terminal region (Fig 2, upper panels, lanes 4). The lower bands detected by anti-LiPABPs antibodies may result from cleavage of the proteins expressed in the mammalian cells (Fig 2, upper panels, lanes 3). Similarly, lower bands observed in the lane 4, upper panel of the Fig 2B, may be related to partially cleaved forms of the recombinant LiPABP2 protein expressed in *E*. *coli*, since they were absent in protein samples purified under denaturant conditions and appeared when the denaturant agent was removed by dialysis against PBS (S2 Fig).

**Fig 3:**
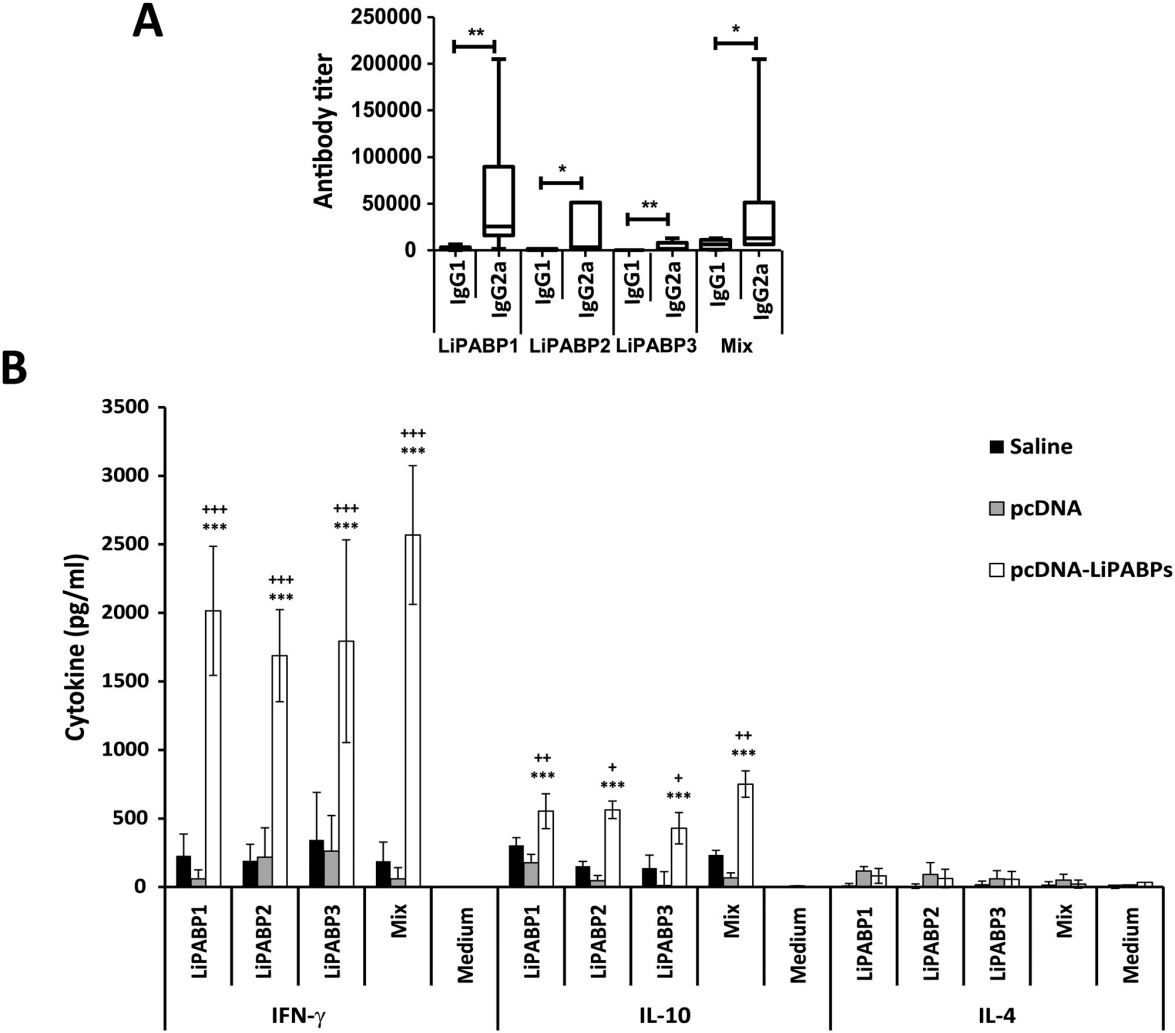
The immunization of the LiPABP genetic combined vaccine induces a predominant Th1-biased response against the LiPABP family in BALB/c mice. Mice (n = 16 per group) were immunized three times with phosphate saline buffer (saline), with the pcDNA3 plasmid or with the LiPABP1 + LiPABP2 + LiPABP3 combined genetic vaccine. The presence of anti—LiPABP1, anti-LiPABP2, anti-LiPABP3 and anti-LiPABPs IgG1 and IgG2a immunoglobulins were individually determined four weeks after the last doses. Sera were assayed from 1/100 to 1/820,000 and horseradish peroxidase-conjugated anti-mouse IgG1 or IgG2a were used as the secondary antibodies at 1/2,000 (A). Results are shown as whisker (min to max) plots. * (*P* < 0.05) and ** (*P* < 0.01) statistical differences between IgG1 and IgG2a reactivity values evaluated by the Mann-Whitney test. At the same time **s**pleen cell cultures were established from eight mice. Splenocytes (5 x 10^6^ cells/ml) were cultured for 72 h at 37°C, 5% CO_2_ in the presence of parasite LiPABP1, LiPABP2 and LiPABP3 as independent stimuli (12 μg/ml) or a mixture of the three proteins (12 μg/ml total protein, 4 μg/ml each one). As background control, parallel cultures were maintained without stimulation (Medium). The levels of IFN-γ, IL-10 and IL-4 were determined by capture ELISA in culture supernatants (B). Each bar represents the mean ± standard deviation (SD) of data taken from eight individual mice. ^+^ *P* < 0.05, ^++^ *P* < 0.01 and ^+++^ *P* < 0.001 significant differences between LiPABP vaccinated and saline control mice; ** *P* < 0.01 and *** *P* < 0.001 significant differences between LiPABP vaccinated mice and pcDNA3 immunized control mice (Student T-test). Results are representative of two independent experiments.

Once determined the correct expression of the three *Leishmania* PABPs, plasmid DNAs were administered to mice as a mixed formulation (combined genetic vaccine). The cellular and humoral responses elicited by the LiPABP combined genetic vaccine were compared to those observed in two control mice groups: one receiving the vaccine saline diluent (PBS) and one receiving the pcDNA3 plasmid (as an adjuvant control). Mice vaccinated with the LiPABP genetic combined vaccine showed a positive IgG response against parasite proteins that was absent in both control groups. An IgG2a versus IgG1 dominant antibody response was observed in vaccinated mice against the antigens composing the vaccine when they were assayed by ELISA, together or separately (Fig 3A). The LiPABP1 protein was the most immunogenic of the three tested LiPABPs in BALB/c mice. No reactivity against SLA was observed in the sera of control or vaccinated mice. The systemic cellular response elicited by the immunizations was studied by the *in vitro* stimulation of spleen cells cultures established from control and LiPABP vaccinated BALB/c mice. A parasite LiPABP-specific secretion of IFN-γ and IL-10 was observed in mice vaccinated with the LiPABP based genetic vaccine. IFN-γ was the most abundant cytokine detected in cultures supernatants after stimulation of cells with each one of the LiPABPs or with a mixture of the three proteins (Fig 3B). Low levels of IL-4 were detected in all cell cultures supernatants assayed. Since the assays were performed in the absence of an IL-4 receptor blocking antibody it cannot be discarded the consumption of the cytokine in supernatants of cells stimulated with the antigens [34]. However, the low titer of LiPABPs specific IgG1 antibodies found in the LiPABP vaccinated mice (an IL-4-dependent isotype [35]) is reflecting the generation of poor Th2 responses. From both humoral and cellular data, it was concluded that the mixed genetic vaccine induced a predominant Th1-biased immune response specific for the three members of the LiPABP family.

### Vaccination of mice with the genetic LiPABP-based combined vaccine protects BALB/c mice against *L*. *major* challenge

**Fig 4:**
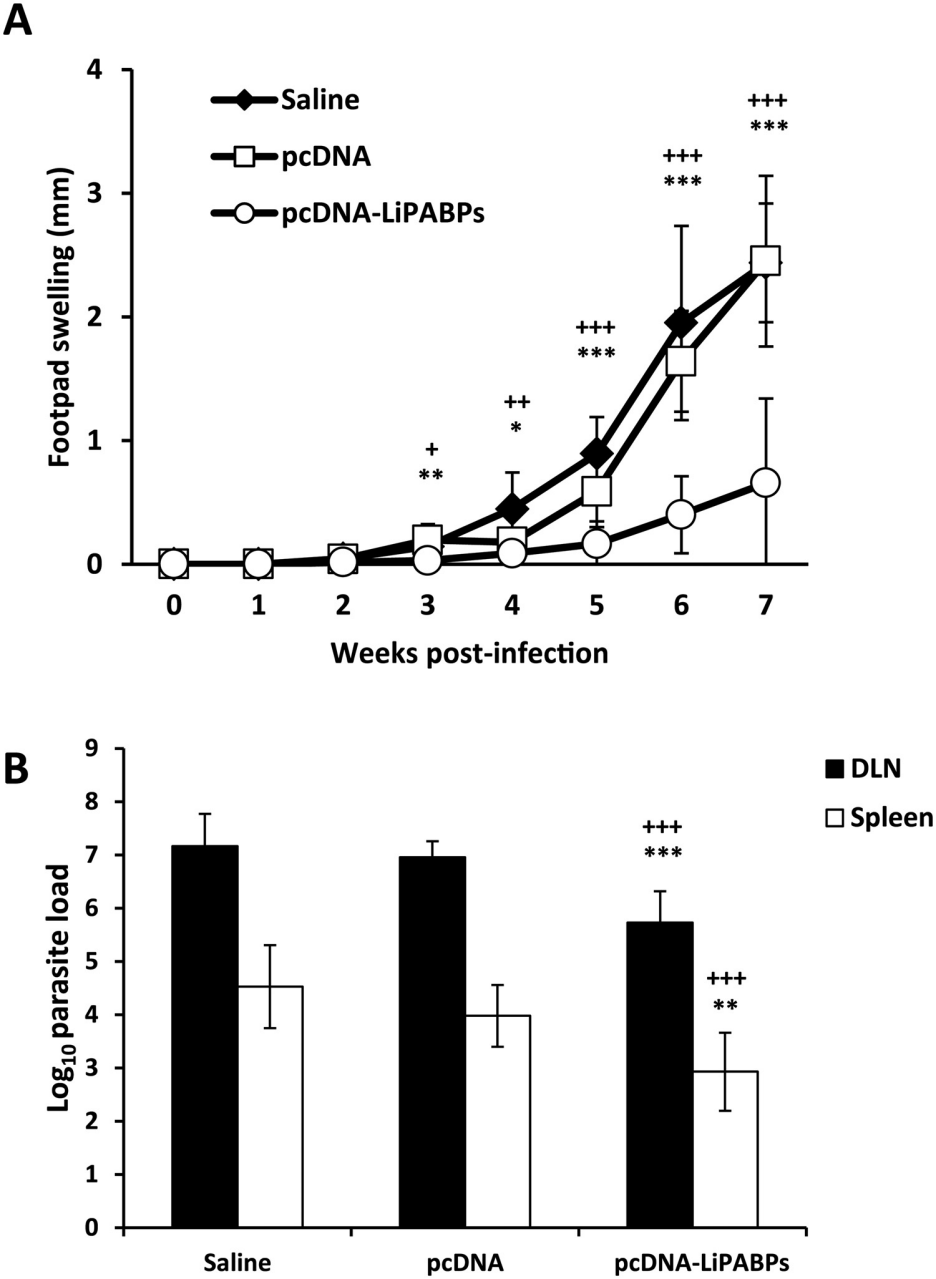
Course of *L*. *major* infection in BALB/c mice vaccinated with the LiPABP DNA combined vaccine. Mice (n = 8 per group) vaccinated with the LiPABP1 + LiPABP2 + LiPABP3 combined genetic vaccine (pcDNA3-PABPs) or inoculated with the pcDNA3 empty vector (pcDNA) or receiving the vaccine diluent (Saline) were challenged in the footpad with 5 × 10^4^ *L*. *major* stationary promastigotes one month after the last immunization. Footpad swelling is represented as the mean ± SD of the difference of thickness between the infected and the uninfected contra-lateral footpads (A). The numbers of viable parasites in the popliteal lymph nodes draining the infected legs (DLN) or in spleens were individually determined by limiting dilution at week seven post-challenge. Mean ± SD of the parasite burdens in the complete organ is shown (B). ^+^ *P* < 0.05, ^++^ *P* < 0.01 and ^+++^ *P* < 0.001 significant differences between LiPABP vaccinated and saline control mice; * *P* < 0.05, ** *P* < 0.01 and *** *P* < 0.001 significant differences between LiPABP vaccinated mice and pcDNA3 immunized control mice (Student T-test). Results in each panel are representative of two independent experiments.

Since the induction of a Th1 response against different parasite antigens has been related with protection in experimental models of CL we next analyzed whether the immunization with the combined vaccine was able to induce protection against *L*. *major* infection in the highly susceptible BALB/c strain. For that purpose, mice vaccinated with the LiPABP based genetic vaccine as well as mice from control groups (inoculated with the non-recombinant plasmid or the saline diluent of the vaccine) were infected with *L*. *major*, one month after the last immunization. Mice vaccinated with the LiPABP combined vaccine showed lower footpad swelling than control groups inoculated with the vaccine diluent or the non-recombinant plasmid, from week 3 after challenge to the end of the assay (Fig 4A). The time for euthanization, seven weeks post-infection, was determined by the appearance of necrotic lesions in control groups that were absent in the LiPABP vaccinated group. In addition, LiPABP vaccinated mice showed a 1.25-log and 1.21-log reduction in the parasite burdens of the lymph node draining the infected footpad (popliteal lymph node) relative to saline and pcDNA3 immunized control groups, respectively (Fig 4B). Similarly, it was determined the existence of a 1.5-log and 1.4-log reduction in the number of viable parasite detected in the spleen of LiPABP vaccinated mice relative to both control groups (Fig 4B).

**Fig 5:**
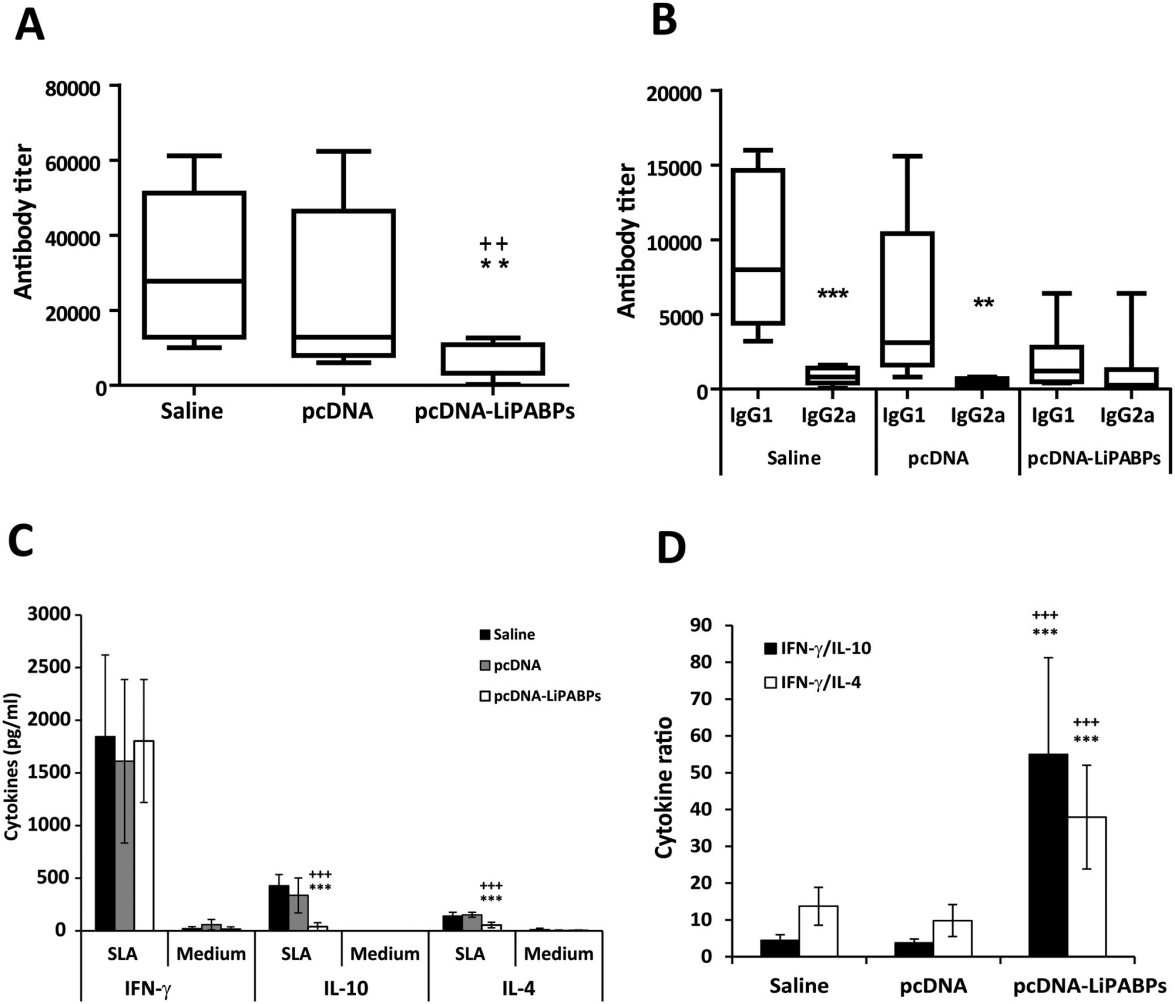
Antibody titers and cytokines production against SLA after infection in control and vaccinated mice groups. The humoral and cellular response against soluble *Leishmania* antigens (SLA) was determined at week seven after *L*. *major* infection in mice (n = 8 per group) previously vaccinated with the LiPABP1 + LiPABP2 + LiPABP3 combined genetic vaccine (pcDNA3-PABPs) or immunized with the pcDNA3 empty vector (pcDNA) or that received the vaccine diluent (Saline) prior to infection. Anti—SLA IgG (A) or IgG1 and IgG2a (B) titers were individually determined by assaying sera from 1/50 to 1/820,000 and using a horseradish peroxidase-conjugated anti-mouse IgG, IgG1 or IgG2a secondary antibody at 1/2,000 dilution. Results are shown as whisker (min to max) plots. For cytokine determinations spleen cells were cultured for 72 h at 37°C, 5% CO_2_ in the absence or in the presence or soluble *Leishmania* antigen (SLA; 12 μg/ml). Levels of IFN-γ, IL-10 and IL-4 were assessed by ELISA in culture supernatants (C). Each bar represents the mean ± standard deviation (SD). The IFN-γ/IL-10 and IFN-γ/IL-4 ratios were determined and represented as the mean ± standard deviation (SD) (D). In (A) ^++^ (*P* < 0.01) and ** (*P* < 0.01) statistical decrease in anti-SLA response in the LiPABPs vaccinated group with respect saline or pcDNA3 control groups, respectively. In (B) ** (*P* < 0.01) and *** (*P* < 0.001) statistical differences between IgG1 and IgG2a anti-SLA titers within each group (Mann-Whitney test). In (C and D) ^+++^ *P* < 0.001 and *** *P* < 0.001 significant differences between LiPABP vaccinated and saline and pcDNA3 control groups, respectively (Student T-test). Results are representative of two independent experiments.

Since the level of the humoral responses elicited against leishmanial antigens in mice infected with *L*. *major* has been correlated to the severity of the disease [36] the humoral response against SLA was analyzed in the three mice groups assayed. At week seven after infection the titer of SLA-specific IgG antibodies in the LiPABP vaccinated mice group was lower than the titer observed in the non-protected control groups (Fig 5A). In addition, the polarization towards a Th2 response demonstrated by titers of SLA-specific IgG1 antibodies observed in both control groups is absent in the LiPABP vaccinated group (Fig 5B). The cellular response against SLA was then analyzed using spleen cells cultures established from mice from the three tested groups. A significant decrease in the production of SLA-induced IL-10 and IL-4 was observed in the LiPABP vaccinated and protected mice, relative to both control groups (Fig 5C). Even though similar levels of IFN-γ were found in the supernatants of cultures after stimulation with SLA the ratio of IFN-γ/IL-10 and IFN-γ/IL-4 was significantly higher in the protected mice group than in animals from both control groups (Fig 5D). As control, spleen primary cultures were grown in the same conditions but in the absence of any other stimuli. The cytokine levels found in these supernatants were very low (Fig 5C; Medium). Therefore, the parasites colonizing the spleen of infected mice were not contributing to cytokine release in the *in vitro* stimulation assay.

It was concluded that immunization with the LiPABPs combined vaccine induced a delay in the progression of CL due to *L*. *major* infection. This partial protection was correlated to the decrease of parasite specific IL-4 and IL-10 mediated responses, in the absence of changes in the magnitude of the parasite dependent IFN-γ production.

### Administration of the genetic combined vaccine was able to switch the immune response elicited against the LiPABP family after infection

The immune response against the LiPABPs after challenge was studied in mice vaccinated with the LiPABP genetic vaccine as well as in mice without a previous contact with these antigens, namely saline and pcDNA3 to analyze how infection affects the immune response elicited against the proteins composing the LiPABP vaccine in immunized mice, but also in animals without a previous contact with these proteins (saline and pcDNA3 groups).

**Fig 6:**
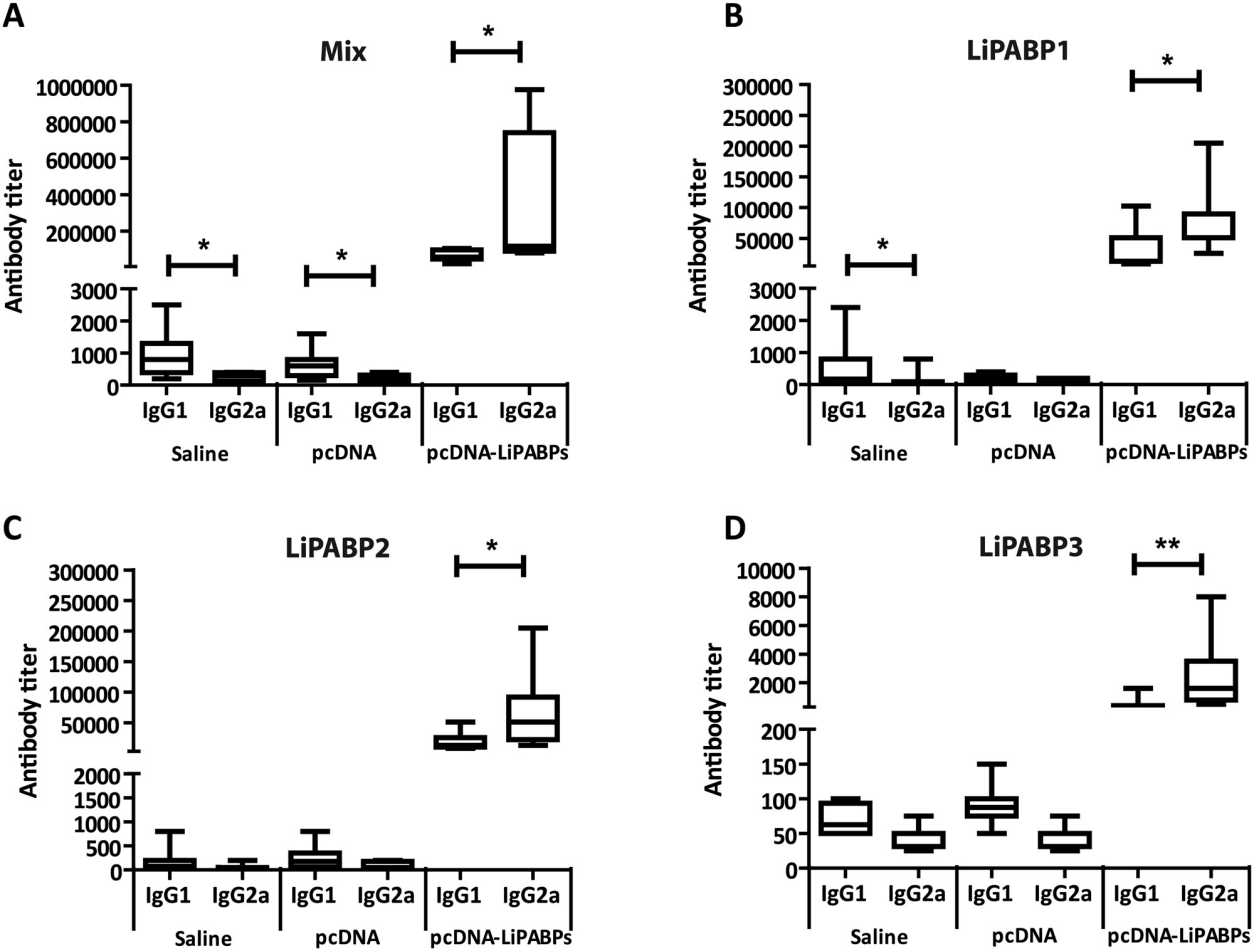
Analysis of the humoral response induced against the LiPABPs after challenge. IgG1 and IgG2a antibody titers specific for a mix of the LiPABPs (A; Mix), LiPABP1 (B), LiPABP2 (C) and LiPABP3 (D) were individually determined by ELISA in mice (n = 8) immunized with a mixture of pcDNA3 plasmids encoding the LiPABP1, LiPABP2 and LiPABP3 proteins (pcDNA-LiPABPs), or controls groups inoculated with saline (Saline) or immunized with pcDNA3 vector (pcDNA) and challenged with *L*. *major* one month after last immunization. Prior to titer determination, sera were assayed at 1/200 dilution to monitor the reactivity against the LiPABP proteins. For sera showing moderate reactivity titers were determined from 1/50 to 1/12,8000. For sera showing high reactivity, titers were determined from 1/500 to 1/1,024,000. Horseradish peroxidase-conjugated anti-mouse IgG1 or IgG2a were used as the secondary antibodies at 1/2,000. Results are shown as whisker (min to max) plots. * (*P* < 0.05) and ** (*P* < 0.01) statistical differences between IgG1 and IgG2a reactivity values evaluated by the Mann-Whitney test. Results of each panel are representative of two independent experiments.

Regarding the humoral response, high IgG1 and IgG2a antibody titers were observed in the sera from LiPABP vaccinated animals after infection (Fig 6A). The predominant IgG2a response against the LiPABP family induced by vaccination was maintained after parasite challenge in this mice group (Fig 6A). The LiPABP1 (Fig 6B) was the most antigenic member of the family followed by LiPABP2 (Fig 6C), being LiPABP3 the lesser antigenic member of the family (Fig 6D). On the contrary, control groups showed a limited humoral response against the LiPABPs antigens, being the IgG1 titers higher than the IgG2a ones (Fig 6A–6D). Significant differences between both subclasses were only observed in both control groups when a mix of the three proteins were employed for coating ELISA plates (Fig 6A) or in the saline group for *Leishmania* LiPABP1 (Fig 6B).

**Fig 7:**
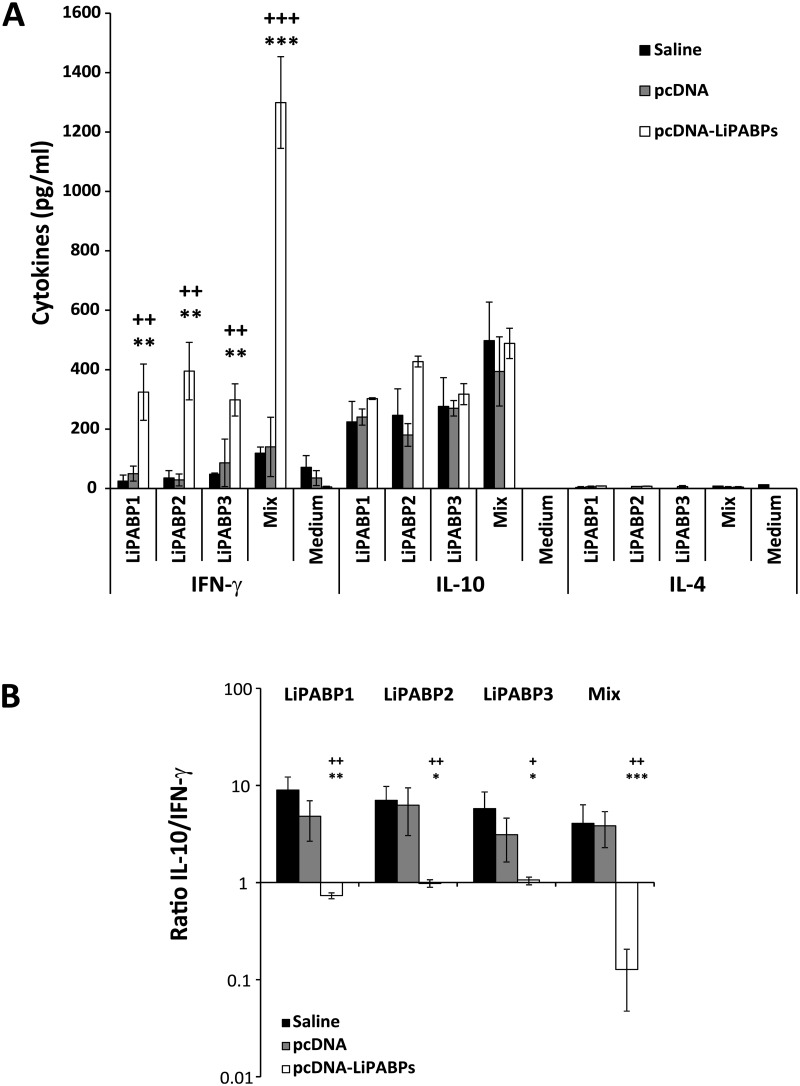
Cytokine production against the LiPABPs after infection in control and vaccinated mice groups. Spleen cell cultures from mice that received saline (Saline), or that were immunized with pcDNA3 plasmid (pcDNA) or with the LiPABP1 + LiPABP2 + LiPABP3 combined genetic vaccine (pcDNA-LiPABPs) were established at week seven after challenge with *L*. *major* (n = 8 per group). Splenocytes (5 x 10^6^ cells/ml) were cultured for 72 h at 37°C, 5% CO_2_ in the presence or parasite LiPABP1, LiPABP2 and LiPABP3 as independent stimuli (12 μg/ml), or with a mixture of the three proteins (12 μg/ml total protein, 4 μg/ml each one). As background control, parallel cultures were maintained without stimulation (Medium). Levels of IFN-γ, IL-10 and IL-4 were assessed by ELISA in culture supernatants. Each bar represents the mean ± standard deviation (SD) (A). The IL-10/ IFN-γ ratio from data shown in panel A was calculated and represented as the mean ± standard deviation (SD) (B). ^+^ *P* < 0.05, ^++^ *P* < 0.01, ^+++^ *P* < 0.001 or; * *P* < 0.05, ** *P* < 0.01, ***P < 0.001 significant differences between LiPABP vaccinated and saline or pcDNA3 control mice groups, respectively (Student T-test). Results are representative of two independent experiments.

The cellular response induced against the LiPABPs by infection was also analyzed in the three mice groups after challenge. An IFN-γ mediated response was only observed in the animals immunized with the LiPABP genetic combined vaccine (Fig 7A). Similar levels of IL-4 and IL-10 specific for LiPABPs were observed in all mice groups (Fig 7A). Very low levels of IL-4 were observed in supernatants of cultures stimulated with the recombinant LiPABPs; less than 20 pg/ml for all groups and antigens tested. Consumption of the cytokine in supernatants of cells stimulated with the antigens can-not be discarded, since the assays were performed in the absence of an IL-4 receptor blocking antibody. A robust IL-10 mediated response was observed against the LiPABPs proteins. At week seven after infection, the three proteins were able to induce the secretion of IL-10 in the spleen cells cultures established from mice belonging to both control groups (Fig 7A). The higher values of the IL-10/ IFN-γ ratio obtained in control groups (Fig 7B) suggest that the members of the LiPABP family may be acting as an anti-inflammatory stimulus during infection. The lower value of the IL-10/ IFN-γ ratio observed in the LiPABP vaccinated and protected mice after infection indicates that the Th1 response induced by vaccination with the genetic combined vaccine against the three member of the LiPABP family was maintained after infection (Fig 7B). Very low levels of the different cytokines were found in cultures grown without stimulation (Fig 7A; Medium).

## Discussion

Human and canine VL patients present high titers of antibodies against different parasite antigens. Many of them are proteins with an intracellular location (like cytosolic or nuclear factors) having a physiological role inside the cell. These antigenic molecules have been named panantigens, because of their conserved nature and their antigenicity in different forms of leishmaniasis as well as in other autoimmune or infectious diseases [37,38]. Alternatively, they are termed pathoantigens [39], because the antibodies produced against them during infection seem to be involved in the development of pathology in the visceral forms of the disease [36,40]. Data shown in this work indicate that the LiPABP family can be included within these groups. They are antigenic in human and canine VL patients and they have an intracellular location [26]. As occur for other pathoantigenic proteins, LiPABPs have essential roles in the parasite, controlling the RNA processing and translation [26]. Beside the LiPABPs, other RNA binding proteins, like the *Leishmania* PUF family of protein factors have been described as antigenic in natural and experimental VL [41]. Interestingly, other intracellular ribonucleoprotein complexes (like ribosomes or nucleosome forming histones) or components of protein aggregates (like heat shock proteins) were classified within the panantigenic/pathoantigenic family [37,38,39]. The LiPABPs are also recognized by the sera from patients with other clinical manifestations, namely ML human patients infected by *L*. *braziliensis* (Fig 1), as occur with other intracellular parasite proteins families. The presence of antibodies against intracellular proteins in sera samples from patients of CL an ML, as well as in cured VL patients, reflects the strong antigenicity of these families [30,31,42]. Some variability in the recognition of the individual LiPABPs was observed among sera samples from different patients. This is a hallmark of the humoral response found in human or canine patients for most of the characterized *Leishmania* antigenic proteins [43,44]. This variability should be taken into account in the development of vaccines or diagnostics for human and canine leishmaniasis. Regarding the specificity of the humoral response elicited against LiPABPs, in a previous report it was shown that anti-LiPABP2 antibodies purified from VL canine sera did not show cross-reactivity with the human PABP [27]. The molecular basis of the response specificity might be related to the high degree of divergence between LiPABPs and their human orthologues (S3 Fig). In addition, as it is deduced from Fig 2, antibodies elicited against the three LiPABPs in mice immunized with the recombinant versions of the three LiPABPs were not able to recognize the mammalian PABP.

The induction of humoral responses against these proteins in natural leishmaniasis may depend on specific recognition by the B-cell receptor following parasite destruction after lysis of the parasites mediated by the complement [45,46] or through the activity of NETs (Neutrophil Extracellular Traps) [47,48]. The identification of many of the pathoantigenic proteins in the secretome/excretome of *Leishmania* species [49,50] offers an alternative manner of presentation of these antigens to the host immunological system. In this sense, LiPABP2 has been found to be part of the secretome of *L*. *donovani* [50]. As the infection progresses, the release of the pathoantigens will increase due to the increment in the parasite burden with a concomitant induction of humoral responses that correlate with the development of the immunopathology associated with the progressive forms of leishmaniasis [38,39]. An association between the development of clinical symptoms and the induction of humoral responses was reported in longitudinal studies developed in different experimental models of VL in dog and hamster [51,52]. Some of these intracellular proteins have immunological properties able to modulate the type of response against the parasite. As examples, the cytosolic tryparedoxin, a protein that targets B cells to secrete IL-10 and to produce anti-tryparedoxin specific antibodies [53] or the ribosomal protein S3a that induces polyclonal expansion of B cells in the host [54]. Additionally, some nuclear factors like the protein termed Le22 or the histone H2B specifically activate IL-10 secretion in peripheral blood mononuclear cells (PBMCs) from human VL patients [55,56]. In this sense, our results indicate that BALB/c mice infected with *L*. *major* elicited a moderate humoral response (IgG1 polarized) against the three *Leishmania* PABPs (Fig 6). In spite of the low titer found against the LiPABP in mice from control groups (Fig 6), our data allow to determine that LiPABP proteins are antigenic in mice infected with *L*. *major*. Supporting this conclusion, a comparative analysis of data shown in Fig 3 and data shown in Fig 6 demonstrate that infection was able to boost the humoral response elicited against the LiPABP family in mice vaccinated with the LiPABP combined genetic vaccine. In addition, the high value of the IL-10/ IFN-γ ratio observed in mice from control groups after infection when the LiPABPs were employed to stimulate spleen cell cultures (Fig 7) reinforces the pathoantigenic characteristics of these protein factors, since IL-10 mediated responses promote susceptibility in this mice model [11,57].

It has been proposed that down-modulation of IL-10 mediated responses or the Th2-primed antibody production against pathoantigens can be a promising strategy for the development of vaccines [21,55,58]. Supporting this hypothesis, some pathoantigenic molecules like histones H2A and H2B (antigenic nuclear factors recognized by the sera from human VL patients [59] or dogs infected by *L*. *infantum* [60]) have also been implicated in the induction of proliferative responses and IFN-γ production in PBMCs from cured CL patients and from asymptomatic VL patients [61]. In addition, histone H2B has been described to stimulate IFN-γ producing CD4^+^ T from donors who developed a protective immune response against *Leishmania* [62]. The immunization of vaccines based on the pathoantigenic histones has been associated with the induction of protection in the BALB/c-*L*. *major* model when Th1 inducing adjuvants or strategies were employed [22,63]. Other well-known example is the protein termed LACK; leishmanial homolog of mammalian receptor for activated C kinase. Although LACK was characterized as an antigen recognized by a protective CD4^+^ T cell clone (Th1) obtained from a BALB/c mice immunized with a SLA fraction [64], it was later found to be implicated in the induction of the early IL-4 response against *L*. *major* occurring in this susceptible model [65]. Remarkably, co-administration of recombinant LACK with Th1 adjuvants in BALB/c mice was able to redirect the naturally induced Th2 responses after *L*. *major* infection. Thus, recombinant LACK protein administered with interleukin-12 [66] or as a LACK based DNA vaccine [67] protected BALB/c mice against *L*. *major* infection. Further, it was demonstrated that BALB/c rendered tolerant to LACK, as a result of transgenic expression of this molecule in the thymus, were resistant to infection with *L*. *major* and developed a Th1 response after infection [68]. Finally, the use of a intranasal LACK DNA vaccine was able to induce a protective immunity in BALB/c against the infection by *L*. *amazonensis* [69] a New World distributed species able to cause a broad spectrum of clinical manifestations, from cutaneous to visceral leishmaniasis in human patients [70].

Our results show that the inoculation of a genetic vaccine based on the three LiPABP proteins was able to delay the progression of the disease caused by *L*. *major* infection in BALB/c mice by decreasing the footpad swelling and the parasite burdens from LiPABP vaccinated mice relative to control mice group (Fig 4). This experimental model has been employed because it is highly susceptible to infection showing a progressive form of the disease, as occurs in different clinical forms of leishmaniasis in natural hosts [71]. In addition to these clinical or parasitological evidences, LiPABP vaccinated mice showed a limited humoral response against SLA as well as high SLA-dependent ratios of IFN-γ/IL-4 and IFN-γ/IL-10 (Fig 5) that can be considered immunological markers of protection [11,72,73]. The strategy of employing LiPABP eukaryotic expression plasmids for inoculation was selected due to the capacity of these third generation vaccines to promote a Th1 response against the heterologous *Leishmania* proteins expressed in the hosts [74]. Due to the heterogenicity of the immune response elicited against LiPABPs in natural and experimental hosts, we used a combined genetic vaccine, instead of the immunization of plasmids encoding the individual antigens. It is likely that the three LiPABPs may be presented to the immune system at the same time after parasite lysis (for B-cell responses) or presented at the same time by professional antigen presenting cells. Thus, induction of a Th1 response against the three proteins may help to reinforce the capacity of the vaccine in controlling disease progression. Using the same mice model employed in this work, the protective capacity of a vaccine based on histones was reinforced when it was formulated including the four nucleosome histones instead of the use of vaccines based on individual or different pairs of histones [75]. It has been generally demonstrated that experimental vaccines combining different parasite antigens conferred more solid protection than those containing individual parasite determinants (reviewed in [17,74]). Our results demonstrated that genetic vaccination of the LiPABPs combined vaccine in BALB/c mice induced a Th1 type response: anti-LiPABP IgG2a antibodies and IFN-γ secretion upon re-stimulation of spleen cells with the recombinant LiPABPs (Fig 3). The immune response induced by LiPABP vaccination was maintained after infection. The PABPs specific IgG2a/IgG1 and IFN-γ IL-10 ratios were reverted in the LiPABP vaccinated and protected mice, in comparison to mice from both control groups after infection (Figs 6 and 7). Even though SLA is a preparation of total parasite soluble proteins, including the LiPABPs, opposite patterns in SLA specific (Fig 5B) and LiPABPs specific (Fig 6) antibody responses were observed when sera from LiPABP vaccinated and infected mice were employed. These differences may be related to the amounts of the LiPABPs present in the SLA preparation. Thus, ELISA assays performed with the whole antigenic repertoire of the parasite may minimize the reactivity of the anti-LiPABPs antibodies. In this context, the anti-SLA immune response can be considered a marker of the immune humoral response against the whole parasite.

Despite the partial protection afforded by the LiPABP combined vaccine in the BALB/c-*L*. *major* model, the use of these proteins as protective antigens should not be underestimated. Some vaccine candidates inducing partial protection in this mice model have emerged as good candidates when tested in other models of infection. As an example BALB/c mice immunized with a DNA vaccine based on *L*. *infantum* P0 (LiP0) or with the recombinant LiP0 protein combined with a Th1 inducing adjuvant, presented a partial protection after challenge with *L*. *major* [76,77]. LiP0 vaccinated mice showed an initial significant reduction in lesion size after challenge, but mice ultimately developed non-healing lesion. The delay in the onset of cell growth was accompanied by a substantial decrease in the parasite load and was correlated to the generation of initial Th1 responses that were changed to a mixed Th1/Th2 response against the LiP0 when disease progressed. Interestingly, our data showed that the immune response induced by vaccination against the LiPABP family members was not changed after infection. On the other hand, the Th1 responses induced by administration of the LiP0 vaccines conferred protection against CL in C57BL/6 mice [77] or against VL in hamster [78]. Similarly, vaccines based on the *L*. *major* ribosomal protein LmL3 or LmL5 combined with CpG-ODNs in BALB/c mice induced partial protection against CL due to *L*. *major* or *L*. *amazonensis* but protect mice against VL caused by *L*. *infantum* and against murine CL due to a high virulent challenge of *L*. *braziliensis* [19,44]. Remarkably, some other vaccines described as protective in the *L*. *major*-BALB/c model were unable to induce protection in the later model [79]. Data presented here should be taken as a proof of concept regarding the protective characteristics of *Leishmania* PABPs and further assays using alternative forms of administration and different models of experimental infection should be performed to determine the real potential of the PABPs for the development of vaccines. As an example, DNA vaccines based on the LACK antigen were able to induce robust Th1 responses but were unable to protect mice from *L*. *mexicana* [80], *L*. *donovani* [81] or *L*. *chagasi* [82] infection. Notwithstanding, it was demonstrated that the induction of more complex immune responses, using prime boost strategy, combining a DNA vaccine and attenuated viruses (Western reserve virus or vaccinia virus) expressing LACK was correlated to protection against murine VL caused by *L*. *infantum* infection [83]. In this sense, dogs experimentally infected with *L*. *infantum* were protected against VL following an heterologous prime-boost vaccination regime with a DNA vaccine encoding LACK and recombinant vaccinia virus (rVACV) expressing LACK [84], or its corresponding non-replicative modified vaccinia (MVA-LACK) [85]. In addition, combination of LiPABPs with other protective antigens should not be ruled out. Of interest, this work demonstrate that *Leishmania* PABPs are antigenic in different vertebrate hosts infected by distinct parasite species (*L*. *infantum* and *L*. *braziliensis* in human, *L*. *infantum* in dogs and *L*. *major* in mice) and the high degree of *Leishmania* PABPs sequence conservation in different *Leishmania* species (S1 Fig) makes plausible to test their cross-protective properties. The possibility to induce cross-protection by the use of vaccines based in proteins conserved among *Leishmania* species is one of the advantages of this type of vaccines [86].

As conclusion, this work has established for the first time the antigenicity of the LiPABPs in different forms of natural leishmaniasis, as well as in BALB/c mice infected with *L*. *major*. In this model of murine progressive leishmaniasis, the administration of a LiPABPs based vaccine was able to dampen the LiPABP-specific humoral and IL-10 mediated responses detected in the non-vaccinated mice after infection. This control of pathogenic-skewed immune responses correlated to the induction of partial protection. In accordance with previous reports, data presented here indicate that controlling the responses associated with susceptibility and elicited by parasite pathoantigens may be relevant to achieve a protective vaccine against *Leishmania* infection.

### List of accession numbers for genes and proteins mentioned in the text

*Leishmania infantum* PABPs:

LiPABP1 LinJ.35.5360 GeneDB.
LiPABP2 LinJ.35.4200 GeneDB.
LiPABP3 LinJ.25.0080 Gene DB.
*Homo sapiens* PABP:
GenBank: EAX07265.1

## Supporting Information

S1 Fig Sequence identity comparisons among the three PABPs from *Leishmania infantum*, *Leishmania major* and *Leishmania braziliensis*. The identity values between the three LiPABPs as well as the identity values of the comparison among the three parasite species, is shown (determined by the Smith-Waterman local alignment of sequences (http://emboss.bioinformatics.nl/)). The accession numbers are also included.
(TIF)

S2 Fig Stability of the purified LiPABP2 recombinant protein in the presence or in the absence of urea denaturant agent. Coomassie-staining of a 10% SDS-PAGE showing a Molecular Weight Marker (Mr), a total extract of protein from *E*. *coli* cultures expressing the LiPABP2 solubilized under denaturant conditions (20 mM Tris ClH pH 8.0, 0.5 M NaCl, 8 M Urea, 1 mM β-mercaptoethanol) (1), the Ni-NTA flow-through fraction (2), the recombinant LiPABP2 under denaturant conditions (3) and the recombinant LiPABP2 dialyzed against PBS (4).
(TIF)

S3 Fig Amino acid comparison among LiPABPs and human PABP (GenBank accession number EAX07265). The amino acid comparisons, as well as the identity and similarity values, were determined by the Smith-Waterman local alignment of sequences (http://emboss.bioinformatics.nl/). The sequence of the human PABP was rescued as the most identical PABP using the three LiPABPs as probes in a BLAST analysis (http://www.ncbi.nlm.nih.gov/).
(DOCX)
