# Supplementary figures and images for: Coadministration of the Three Antigenic Leishmania infantum Poly (A) Binding Proteins as a DNA Vaccine Induces Protection against Leishmania major Infection in BALB/c Mice
Source: PLoS Negl Trop Dis. 2015 May 8;9(5):e0003751. doi: 10.1371/journal.pntd.0003751 (PMC4425485; doi:10.1371/journal.pntd.0003751)

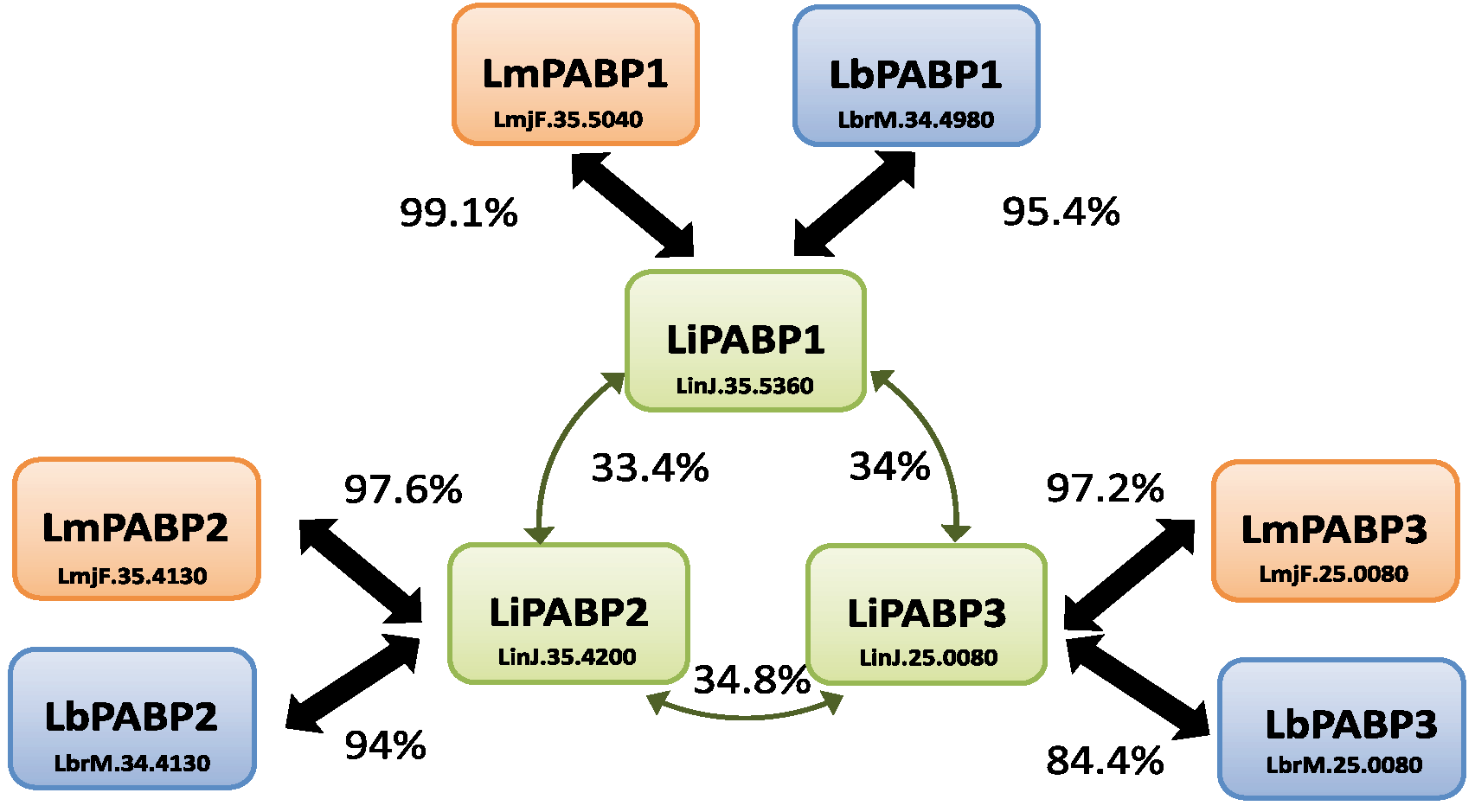

Supplement: S1 Fig — The identity values between the three LiPABPs as well as the identity values of the comparison among the three parasite species, is shown (determined by the Smith-Waterman local alignment of sequences (http://emboss.bioinformatics.nl/)). The accession numbers are also included. (TIF) [file pntd.0003751.s001.tif]

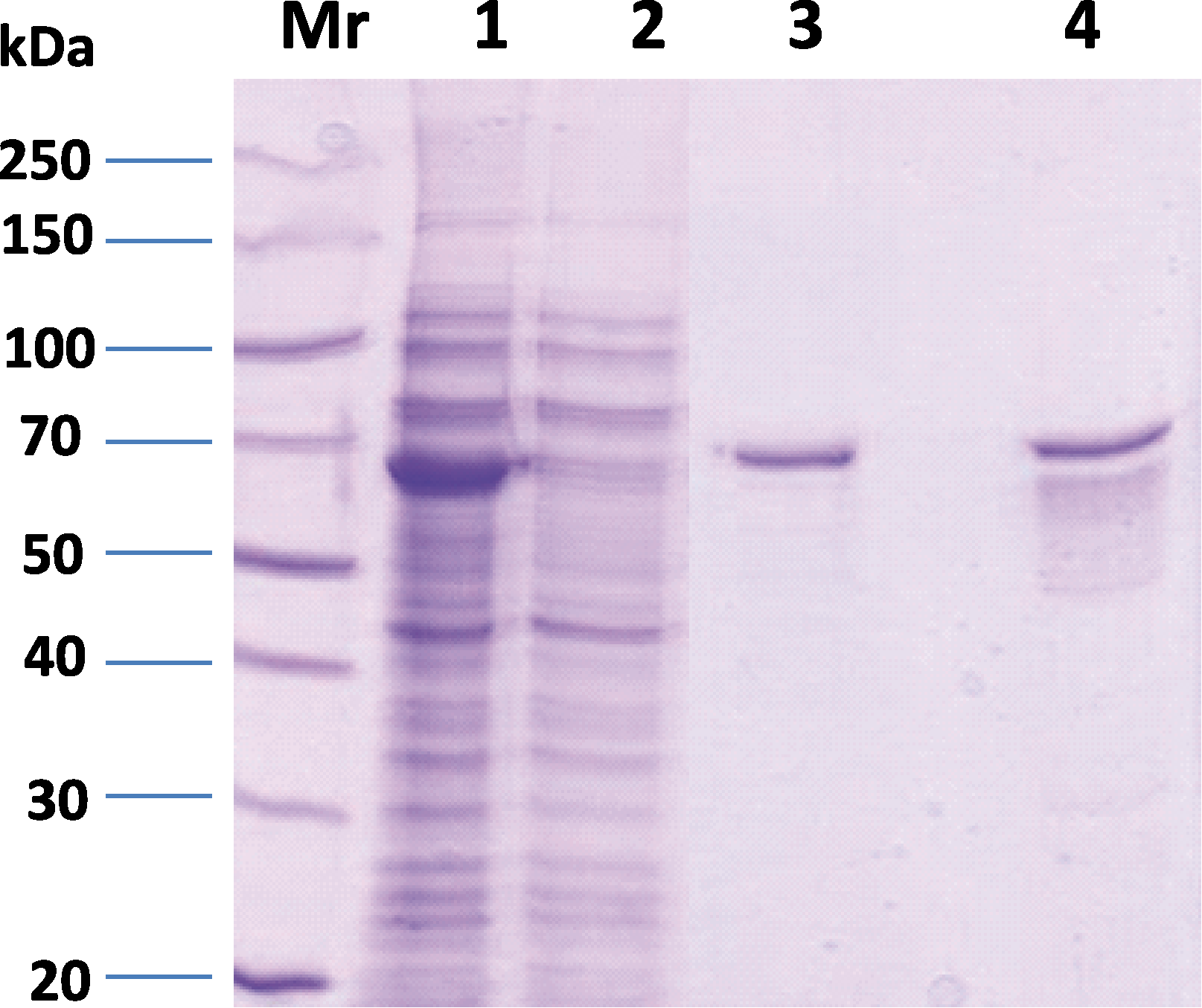

Supplement: S2 Fig — Coomassie-staining of a 10% SDS-PAGE showing a Molecular Weight Marker (Mr), a total extract of protein from E. coli cultures expressing the LiPABP2 solubilized under denaturant conditions (20 mM Tris ClH pH 8.0, 0.5 M NaCl, 8 M Urea, 1 mM β-mercaptoethanol) (1), the Ni-NTA flow-through fraction (2), the recombinant LiPABP2 under denaturant conditions (3) and the recombinant LiPABP2 dialyzed against PBS (4). (TIF) [file pntd.0003751.s002.tif]
