# Supplementary material for: Coadministration of the Three Antigenic Leishmania infantum Poly (A) Binding Proteins as a DNA Vaccine Induces Protection against Leishmania major Infection in BALB/c Mice
Source: PLoS Negl Trop Dis. 2015 May 8;9(5):e0003751. doi: 10.1371/journal.pntd.0003751 (PMC4425485; doi:10.1371/journal.pntd.0003751)
Supplement: S3 Fig — The amino acid comparisons, as well as the identity and similarity values, were determined by the Smith-Waterman local alignment of sequences (http://emboss.bioinformatics.nl/). The sequence of the human PABP was rescued as the most identical PABP using the three LiPABPs as probes in a BLAST analysis (http://www.ncbi.nlm.nih.gov/). (DOCX) [file pntd.0003751.s003.docx]

**Aminoacid comparison between human PABP (EAX07265; lower case) and *Leishmania infantum* PABP1 (upper case).**

Identity: 39.3%

Similarity: 54.4%

9 pm--aslyvgdlhsdvteamlyekfspagpvlsirvcrdmitrrslgyay 56

|| ||:|||||.:.:.|..|.|.|.|.|.:|::|||||:||:|||||.|

4 PMQIASIYVGDLDATINEPQLVELFKPFGTILNVRVCRDIITQRSLGYGY 53

57 vnfqqpadaeraldtmnfdvikgkpirimwsqrdpslrksgvgnvfiknl 106

|||.....||:|:::|||..:..|.:|:||.||||:||.||.||||:|||

54 VNFDSHDSAEKAIESMNFKRVGDKCVRLMWQQRDPALRYSGNGNVFVKNL 103

107 dksidnkalydtfsafgnilsckvvcdeng-skgyafvhfetqeaadkai 155

::.:|:|:|:|.|:.||:||||||:.||.| |:||.||||:.:.:|..||

104 ERDVDSKSLHDIFTKFGSILSCKVMQDEEGKSRGYGFVHFKDETSAKDAI 153

156 ekmngm---llndrk-vfvgrfksrkereaelgakakeftnvyiknfgee 201

.||||. ...|:| ::|..| ..|.|.|.|....|||||||.....

154 VKMNGAADHASEDKKALYVANF---IRRNARLAALVANFTNVYIKQVLPT 200

202 vddeslkelfsqfgktlsvkvmrdpngkskgfgfvsyekhedankaveem 251

|:.|.:::.|::||...|....:|.:|:. |.|.::|||:||.||||.|

201 VNKEVIEKFFAKFGGITSAAACKDKSGRV--FAFCNFEKHDDAVKAVEAM 248

252 ngkeis-----gkiifvgraqkkverqaelkrkfeqlkqerisryqgvnl 296

:...|. |:.::|.|||.:.||...|::|:.| .:..|.||

249 HDHHIDGITAPGEKLYVQRAQPRSERLIALRQKYMQ------HQALGNNL 292

297 yiknlddtiddeklrkefspfgsitsakvml-edgrskgfgfvcfsspee 345

|::|.|.......|.:.|..:|.:.|.:||: |.|.|:|||||.||:.:|

293 YVRNFDPEFTGADLLELFKEYGEVKSCRVMVSESGASRGFGFVSFSNADE 342

346 atkavtemngrivgskplyvalaqrkeerkahltnqymqrvagmralpan 395

|..|:.|||||::..|||.|.:|||:::|...|..|:.||:..|...

343 ANAALREMNGRMLNGKPLIVNIAQRRDQRYTMLRLQFQQRLQMMMRQ--- 389

396 ailnqfqpaaggyfvpavpqaqgrppyytpnqlaqmr-pnprwqqggrpq 444

::|..|..|| |||| || ...|.|.|||.|

390 --MHQPMPFVGG---------QGRP----------MRGRGGRQQLGGRAQ 418

445 gfqgmpsairqsgprptlrhlaptgvptavqnlapraavaaaapravapy 494

|. |..:|:..|..||......|.|.||.

419 GH--------------------PMPMPSPQQPQAPAQPQGFATPSAVG-- 446

495 kyassvrsphpaiqplqapqpavhvqgqepltasmlaaappqeqkqmlge 544

:..:.....|...|...|.| |:|...|.:..||||:..||:

447 -FVQATPKHSPGDVPETPPLP--------PITPQELESMSPQEQRAALGD 487

545 rlfpliqtmhsnlagkitgmlleidnsellhmlespeslrskvdeavavl 594

|||..:..:...||.|||||.||:...|...:|...:.|..:|.||:.||

488 RLFLKVYEIAPELAPKITGMFLEMKPKEAYELLNDQKRLEERVTEALCVL 537

595 qah 597

:||

538 KAH 540

**Aminoacid comparison between human PABP (EAX07265; lower case) and *Leishmania infantum* PABP2 (upper case).**

Identity: 36.9%

Similarity: 53.2%

9 pmaslyvgdlhsdvteamlyekfspagpvlsirvcrdmitrrslgyayvn 58

|..|::||.|..|:.|..|::.|...|||.|:|||.|..|::||||.|||

6 PNPSIWVGGLDPDLQEQKLHDYFVRIGPVTSVRVCVDSATQKSLGYGYVN 55

59 fqqpadaeraldtmnfdvikgkpirimwsqrdpslrksgvgnvfiknldk 108

||.|||||:|||... ..:..:.:||...|||||.|:|||.|:.:|.|.|

56 FQDPADAEKALDQAG-SKLGSRYLRIAKIQRDPSKRRSGVNNILVKKLPK 104

109 sidnkalydtfsafgnilsckvvcdeng-skgyafvhfetqeaadkaiek 157

|:|..||.:.||.||.:.:..:.|||.| |:|||.:.||.:|:|..|:.:

105 SVDTYALKEMFSKFGRLTAIGLACDEKGESRGYARISFEREESAVDAVRE 154

158 mngmllndrkvfvgrfksrkereaelgakakeftnvyiknfgeevddesl 207

|:||.::.:.:.|.|::::...|. .|:|||:|:||....|.||.|

155 MDGMEMDGQAIVVERYQAQHRDEL-----LKQFTNLYVKNLDPAVTDEKL 199

208 kelfsqfgktlsvkvmrdpng--kskgfgfvsyekhedankaveemngke 255

:..|:::|:..|.|| ||... ...|.|:|:::|||:|.:||||:||||

200 RAFFAKYGEVSSAKV-RDLGAVQSEAGLGYVAFQKHENAARAVEELNGKE 248

256 i----sgkiifvgraqkkverqaelkrkfeqlkqerisryqgvnlyiknl 301

. :|..:.|.|.:.:.|||.:.:|:..:..|:. |:|. |||:|..

249 CEIAKAGSPLDVSRFRSREERQRDRERQRRERAQQH-SKYP--NLYVKGF 295

302 ddtiddeklrkefspfgsitsakvml--edgrskgfgfvcfsspeeatka 349

|||:..|:|.:.|..:|...|..||: |.|.|:.||||.......|::|

296 DDTVTSERLEELFQRYGETVSVTVMMDKETGVSRCFGFVSMKDQNAASQA 345

350 vtemng-rivgskplyvalaqrkeerkahltnqymq-rv-------agmr 390

:.|:|| ..:..:||:|..|.||:.|:.:|..:..| || .||.

346 IQELNGSTFLSPRPLFVTYALRKDARRQNLEERSKQFRVRQNPMGGPGMG 395

391 alpanail--nqfqpaaggyfvpavp--qaqgrppyytpnqlaqmrpnpr 436

|:|....: ..|......:..|.|| ...|.....:.|.:..|.....

396 AMPPIGFMGPQMFNNVNMPFMNPRVPIMPMNGMNGIGSMNGMGGMNGVGG 445

437 wqqggrpqgfqgmpsairqsgprptlrhlaptgvptavqnlapraavaaa 486

....|...|..||.......|.....|.:||..:

446 VGNMGGMGGMGGMGGMGGMGGMGGMARPMAPNAM---------------- 479

487 apravapykyassvrs-phpaiqplqapqpavh----vqgqepltasmla 531

|.:|| |.|...|:|:..|..| .|||. .|::||

480 -----------SQMRSRPMPQKPPMQSLMPQQHQQAPPQGQN--LAAVLA 516

532 aappqeqkqmlgerlfpliqtmhsnlagkitgmlleidnsellhmlespe 581

...|::||.:|||||:..|...|.::|.||||||||:||||:|:||:||.

517 NLNPEQQKNVLGERLYSYIVRSHPSVAAKITGMLLEMDNSEILNMLDSPT 566

582 slrskvdeavavlqah 597

.|.||:.||..||..|

567 MLDSKIAEAQDVLNRH 582

**Aminoacid comparison between human PABP (EAX07265; lower case) and *Leishmania infantum* PABP3 (upper case).**

Identity: 38.1%

Similarity: 51.1%

12 slyvgdlhsdv--teamlyekfspagpvlsirvcrdmitrrslgyayvnf 59

|:|||||..|: .|..:...||...||:|::|||||.|:|||||.||||

9 SVYVGDLPIDLPRPEEAINNLFSTVAPVVSVKVCRDMATQRSLGYGYVNF 58

60 qqpadaeraldtmnf-dvikgkpirimwsqrdpslrksgvgnvfiknldk 108

|..||||:.:|.:|: .:..|:.||:|:|.|||..||||:.|||:|.||.

59 QTTADAEKVIDALNYTGIAPGRQIRVMFSIRDPLQRKSGMNNVFVKKLDA 108

109 sidnkalydtfsafgnilsckvvcdeng-skgyafvhfetqeaadkaiek 157

:|:.|.|...||..|.:|||||..|..| ||||.||.|||.:.|..|::

109 AINAKELQAAFSKCGRVLSCKVALDSAGNSKGYGFVQFETADGAKAALD- 157

158 mngmllndrkvfvgrfksrkereaelgakakeftnvyiknfgeevddesl 207

|||..|.|.:|.|..|..|.:||. ..||.|.|:||||......:..:

158 MNGSKLGDSEVVVAPFVRRVDREV---MAAKSFRNIYIKNITAAATEADV 204

208 kelfsqfgktlsvkvmrdpngkskgfgfvsyekhedankaveemngkeis 257

|....:|||..|:.:.......:| |..|::|:||.|.:|:..:|..|.|

205 KATVEEFGKVDSLFLSEHARFPTK-FALVAFEEHEAAVQAIAALNESEES 253

258 g------kiifvgraqkkverqaelkrkfeqlkqerisryq--gvnlyik 299

| |:: |.||..|.||..| |::..|.|| |.|||:|

254 GLTEKAAKLV-VCRALSKSERDRE--------KKKAASLYQNHGRNLYVK 294

300 nlddtiddeklrkefspfgsitsakvmle-dgrskgfgfvcfsspeeatk 348

:|.|.|.|.|||:.|.|||.|||..:|.| :|..|||.||||...:.|:.

295 HLPDDITDNKLREIFEPFGKITSCAIMKEPNGTLKGFAFVCFEDKQHASA 344

349 avtemngriv--gskplyvalaqrkeerkahltnqymqrvagmr-----a 391

|:..:||..: .:|||||:.|::|:.|...| .||.|.|| |

345 ALRSLNGHPLEHSAKPLYVSHAEQKDMRIRLL----QQRRAAMRHQSRMA 390

392 lpanailnqfqpaaggyfvpavpqaqgrppyytpnqlaqmrpnprwqqgg 441

.|.|.. ||...|.|: |:.:..|.|.|

391 PPMNTF----------------PQQWPRHPF--PHMVPPMMPPP------ 416

442 rpqgfqgmpsairqsgp---rptlrhlaptgvptavqnlapraavaaaap 488

|....|||..: ||| ||.:......|.|

417 -PPPNMGMPQFM--SGPMIRRPVMEPHLMQGEP----------------- 446

489 ravapykyassvrsphpaiqplqapqpavhvqgqepltasmlaaappqeq 538

:|.|:...||.:...|. |.|:.:..:.|:...|::|

447 -----------MRPPNRYTQPREQYPPQ---QRQDGVDMNYLSTLSPEQQ 482

539 kqmlgerlfpliqtmhsnlagkitgmlleidnsellhmlespeslrskvd 588

|..|||.|:..|..:.|:.|.||||||||:...|:..:|....:|.||:.

483 KNYLGELLYSRILPLESSNAAKITGMLLEMSREEIFEILADHFALLSKIQ 532

589 eavavlqah 597

||.||||.|

533 EANAVLQQH 541
